# Supplementary material for: Direct observation and impact of co-segregated atoms in magnesium having multiple alloying elements
Source: Nat Commun. 2019 Jul 19;10:3243. doi: 10.1038/s41467-019-10921-7 (PMC6642188; doi:10.1038/s41467-019-10921-7)
Supplement: Supplementary file 1 — Supplementary Information [file 41467_2019_10921_MOESM1_ESM.pdf]

Direct observation and impact of co-segregated atoms in magnesium having multiple alloying elements

Zhao et al

Supplementary Figures

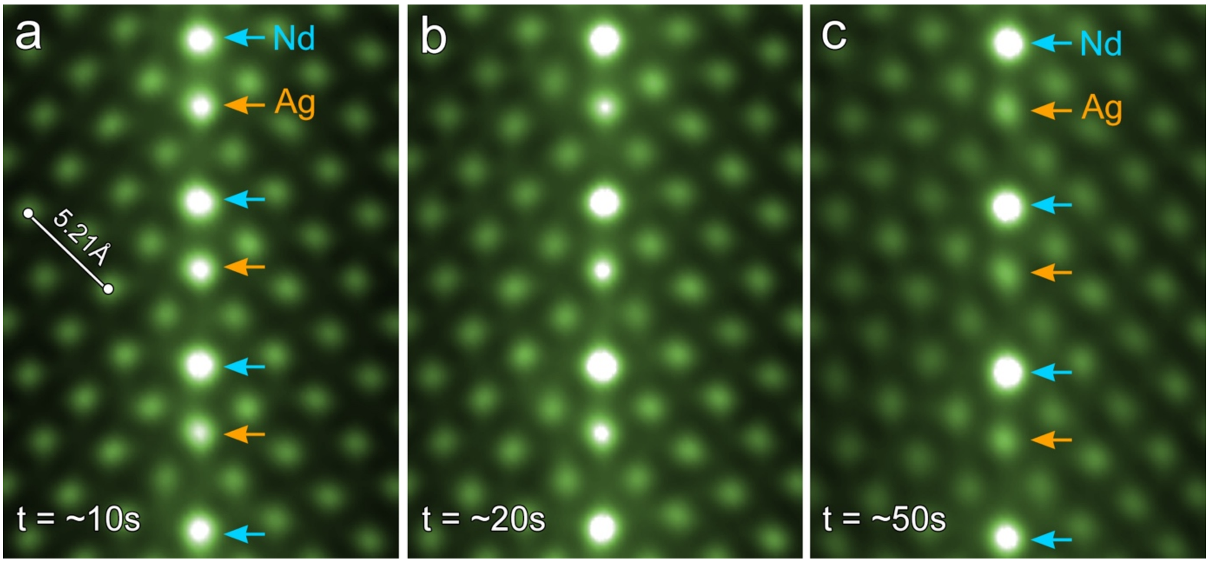

Supplementary Figure 1. Thermal stability of Nd-rich and Ag-rich columns in a coherent (10$\bar{1}$2) twin boundary. (a-c) A series of HAADF-STEM images showing thermal stability of Nd-rich and Ag-rich columns in a coherent (10$\bar{1}$2) twin boundary under continuous electron radiation for different scanning times (given in each image). Electron beam is parallel to the [$\bar{1}$2$\bar{1}$0] direction. Accelerating voltage of electrons is 300 kV.

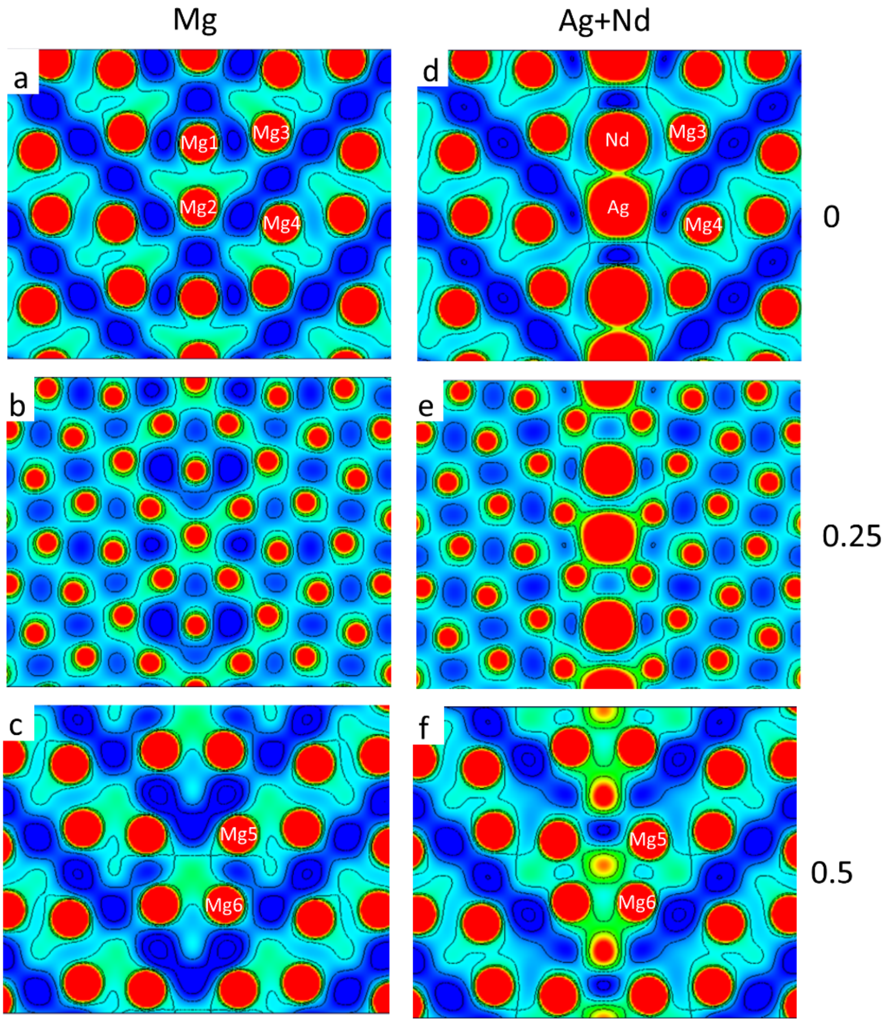

Supplementary Figure 2. Charge density plots for a $(10\bar{1}2)$ twin boundary. Expansion and compression sites are filled with Mg (a-c) and in (d-f) Nd sits in the expansion and Ag in the compression site. A charge density slice in the plane containing the compression and expansion sites is given in (a) and (d). (b) and (e) is a plane ¼ of a cell above or below, and (c) and (f) is a plane ½ of a cell above or below. Without solute segregation, the Mg1 sits in the expansion site of the $(10\bar{1}2)$ boundary and has a calculated Mg1-Mg5 bond distance of 3.31 Å, a Mg1-Mg6 distance of 3.24 Å, and a Mg1-Mg3 distance of 3.58 Å. Mg2 sits in the compression site and has calculated atom distances of 3.06 Å (Mg2-Mg5), 3.10 Å (Mg2-Mg6) 4.12 Å (Mg2-Mg4). These distances can be compared with bulk Mg, where the distances to neighbouring atoms are 3.17 Å and 3.19 Å. When Nd is substituted for Mg in bulk Mg, the Mg lattice is distorted and calculations give distances from Nd to neighbouring Mg atoms at 3.29 Å and 3.31 Å, larger than the equivalent distances in bulk Mg, and so the expansion site at the twin boundary is an ideal location for the Nd to minimise elastic distortion. When incorporated at the expansion site of the twin, the Nd-Mg atom distances are calculated to be 3.37 Å (Nd-Mg6), 3.45 Å (Nd-Mg5), and 3.44 Å (Nd-Mg3). Similarly, when Ag is substituted for Mg in the bulk, the distances from Ag to the neighbouring Mg atoms is calculated to be 3.11 and 3.13 Å, which makes the compression site a location where the strain caused by the Ag can be minimised. When Ag is placed at the compression site the Ag-Mg distances are calculated to be 2.93 Å (Ag-Mg6) and 2.98 Å (Ag-Mg5).
